# Persistent Nausea and Gastrointestinal Distention: A Case Report of Aerophagia

**DOI:** 10.7759/cureus.50070

**Published:** 2023-12-06

**Authors:** Tsutomu Nishida, Yu Higaki, Kenji Watabe

**Affiliations:** 1 Department of Gastroenterology, Toyonaka Municipal Hospital, Toyonaka, JPN; 2 Department of Gastroenterology and Hepatology, Osaka University Graduate School of Medicine, Suita, JPN

**Keywords:** intestinal pseudo-obstruction, mr enterography, nausea, gastrointestinal distention, aerophagia

## Abstract

A 43-year-old woman experienced acute nausea, diarrhea, and abdominal pain, leading her to our hospital. No relevant medical history or physical abnormalities were noted. Symptoms persisted for a month, causing weight loss and abdominal bloating. CT scans revealed distension throughout the gastrointestinal tract without stenosis. Intestinal pseudo-obstruction and aerophagia were suspected. MR enterography confirmed normal gastric and intestinal motility, diagnosing the condition as aerophagia-induced gastrointestinal distention. This case underscores the value of MR enterography in assessing intestinal motility and differentiating between intestinal pseudo-obstruction and aerophagia.

## Introduction

Aerophagia is a functional disorder of the upper gastrointestinal tract. While common in children, adult-onset severe symptoms in the lower intestinal tract are rare. The Rome III consensus previously identified aerophagia as the primary cause of belching disorders, linking it to frequent air swallowing [1]. Although aerophagia has traditionally been linked to excessive belching, it has evolved to encompass a broader range of symptoms and has been increasingly recognized as an outdated term for this symptom. The Rome IV diagnostic criteria replaced the dictum that there was “no evidence for organic disease” in all definitions with “after appropriate medical evaluation the symptoms cannot be attributed to another medical condition” [2]. In particular, cases with dilatation of the lower intestinal tract require differentiation of intestinal pseudo-obstruction (IPO).

Despite evolving definitions, aerophagia remains a key consideration in the clinical approach for functional gastrointestinal disorders characterized by excessive regurgitation. Such disorders often have multifactorial etiologies that encompass both physical and psychological elements. Treatment modalities can vary and include behavioral therapies, prokinetic agents, and proton pump inhibitors, depending on the specific underlying causes and patient characteristics. In this case, magnetic resonance (MR) enterography proved to be an appropriate evaluation to negate the intestinal peristalsis characteristic of IPO and to assess bowel movement for the diagnosis of aerophagia. This paper presents a unique case of an adult patient with aerophagia exhibiting severe lower intestinal symptoms unresponsive to standard medical treatment and who was first diagnosed with IPO.

## Case presentation

**Figure 1:**
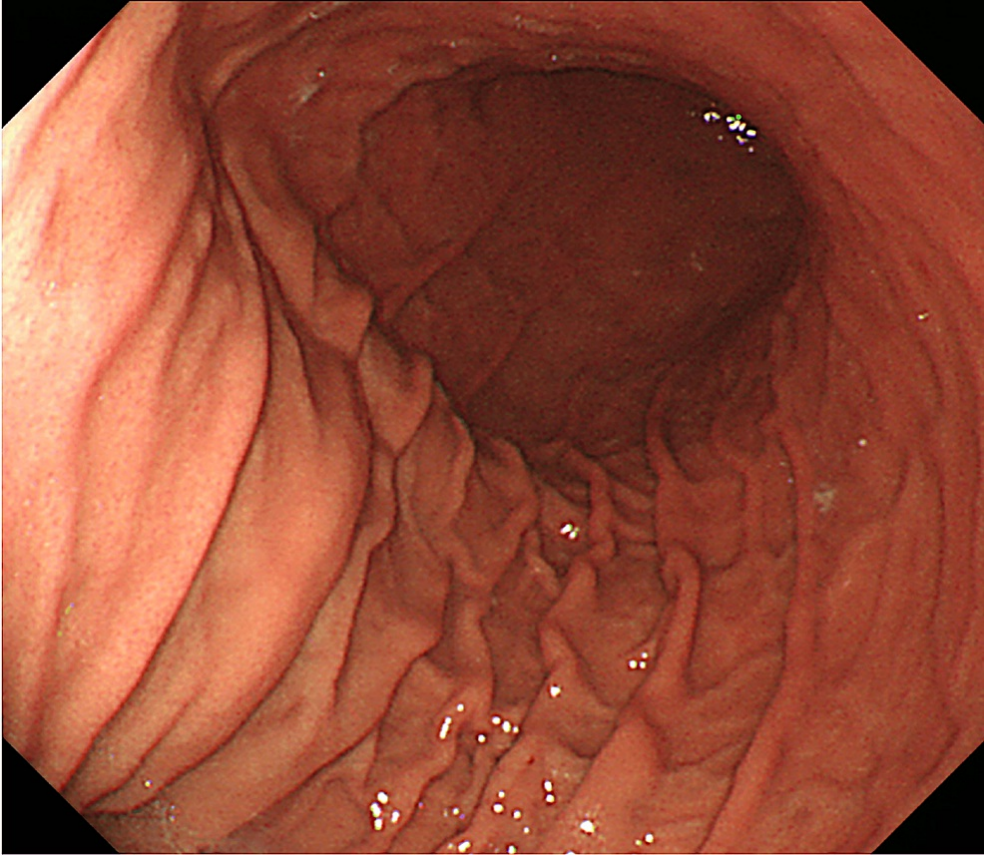
Esophagogastroduodenoscopy image Esophagogastroduodenoscopy indicated a normal mucosal lining without findings of Helicobacter pylori infection.

A 43-year-old woman presented with sudden nausea, diarrhea, and abdominal pain without vomiting. The patient was initially diagnosed with acute gastroenteritis at a local clinic and received medications that alleviated her abdominal pain and diarrhea within a few days. However, nausea persisted. Upon referral to our hospital, esophagogastroduodenoscopy indicated a normal mucosal lining without findings of Helicobacter pylori infection (Figure 1) and displayed regular peristaltic activity without food residue in the stomach.

**Figure 2:**
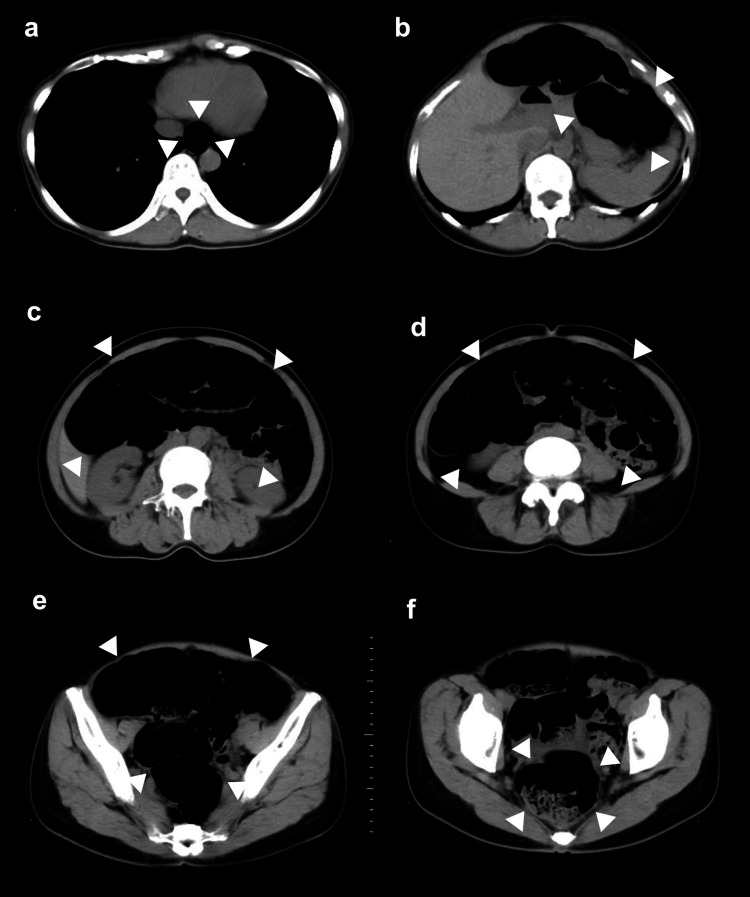
Computed tomography Computed tomography showed no stenosis of the digestive tract and continuous gas filling from the esophagus (Figure 2a, arrow) to the stomach (Figure 2b, arrow), from the small intestine to the colon (Figures 2c, 2d, 2e) and rectum (Figure 2f, arrow).

Further examination of the esophagus and duodenum revealed no abnormalities. The patient had no significant medical history, including abdominal surgery or long-term medication use. Physical examination revealed audible intestinal peristalsis and no weight loss or other abnormalities. Although she reported mild stress, she exhibited no signs of insomnia or psychosomatic disorder. We hypothesized that the patient had functional dyspepsia secondary to acute gastroenteritis. A regimen of potassium-competitive acid blockers and prokinetic medications, including mosapride, acotiamide, and daikenchuto, a widely used Japanese herbal formula, was sequentially administered. Despite these interventions, her symptoms persisted for one month, leading to slight weight loss. Subsequently, the patient developed frequent belching and abdominal distension. Computed tomography (CT) scans indicated a gas-filled gastrointestinal tract extending from the esophagus to the rectum (Figure 2, arrows) without evidence of any stenosis of the gastrointestinal tract (Video 1).

**Video 1:** Computed tomography

We considered acute IPO and aerophagia as possible diagnoses. Colonoscopy showed normal mucosa with regular peristaltic waves and haustra. MR enterography confirmed healthy gastric and small intestinal motility, contradicting the dilated intestines observed on CT scans (Video 2).

**Video 2:** MR enterography

IPO was ruled out because cine MR enterography showed that the small intestine, which was dilated on CT, was moving well. Ultimately, the patient was diagnosed with gastrointestinal distention due to aerophagia. Despite enduring symptoms for 18 months, she experienced dramatic relief following an episode of acute epigastric pain. Follow-up CT revealed reduced gastrointestinal gas extension, and the patient remained symptom-free for 10 months. We concluded that the patient had aerophagia, a rare adult-onset condition that causes severe symptoms such as belching, flatulence, bloating, and abdominal pain. MR enterography was instrumental in evaluating intestinal motility and establishing the diagnosis.

## Discussion

Aerophagia involves excessive air swallowing, causing symptoms like abdominal distension, bloating, regurgitation, and flatulence. While swallowed air is often expelled by belching, it can also contribute to the overall gas content within the gastrointestinal tract. The average individual is estimated to swallow one to two-quarters of air daily, with factors such as smoking, drinking through a straw, and chewing gum potentially exacerbating this amount [3].

Although aerophagia frequently occurs in pediatric populations [4,5], it remains relatively underdiagnosed in adults [6]. A survey by Drossman et al. indicated that although 23% of respondents met the criteria for aerophagia, only 13% sought medical consultation [6]. This disparity may suggest that some adults either manage their symptoms successfully without medical intervention or simply do not find them bothersome enough to seek medical advice. Adult-onset aerophagia with severe symptoms, as demonstrated in the present case, is rare.

Chitkara et al. noted in their study that adults with aerophagia often have longstanding symptoms, with a median duration of two years, primarily comprising belching, bloating, and abdominal distension [3]. In agreement with this, the patient in the current study experienced symptoms for 18 months. Interestingly, while Chitkara et al. categorized aerophagia as a distinct upper functional gastrointestinal disorder, our patient demonstrated distension throughout the gastrointestinal tract, as evidenced by CT imaging [3].

The condition shares symptomatic similarities with IPO, a more severe functional gastrointestinal disorder characterized by symptoms akin to intestinal obstruction but devoid of any mechanical cause. Differentiating aerophagia from IPO is vital for accurate diagnosis and treatment [7].​​​ In MR enterography itself, direct evidence of aerophagia is difficult to obtain. Therefore, in cases with lower intestinal dilatation, such as the present case, it is necessary to distinguish it from IPO and MR enterography is useful in the appropriate evaluation. In this study, MR enterography was employed to evaluate gastrointestinal motility, demonstrating its potential utility in differentiating between these conditions.

MR enterography is increasingly used as a noninvasive tool for assessing gastrointestinal conditions. While its primary use has been in the assessment of inflammatory bowel diseases such as Crohn’s disease [7,8], its potential utility in evaluating gastrointestinal motility, especially in functional gastrointestinal disorders (FGIDs), is an area of growing interest. FGIDs such as irritable bowel syndrome and functional dyspepsia often present with symptoms that are not easily attributable to structural abnormalities. Traditional diagnostic methods, such as endoscopy or radiography, may not provide sufficient insight into the functional aspects of these disorders. MR enterography, with its ability to provide high-resolution images and capture dynamic sequences, offers a more comprehensive view of gastrointestinal motility patterns. MR enterography’s advantage is its noninvasiveness and lack of ionizing radiation, suitable for repeated evaluations. It could identify subtle motility changes that are characteristic of FGIDs, thereby aiding diagnosis and treatment planning. However, the sensitivity, specificity, and cost-effectiveness of the technique in this particular application should be rigorously evaluated in clinical studies. To date, there have been no specific reports of MR enterography evaluations for aerophagia. Very little research has been conducted in this area; therefore, future studies are needed.

## Conclusions

Our case underscores MR enterography’s role in the diagnosis of aerophagia, enriching the limited literature. While this technique may reveal signs such as small bowel dilation, its specific role in diagnosing aerophagia has not been extensively documented. Therefore, this case report contributes significantly to our understanding of the diagnostic and clinical aspects of adult-onset severe aerophagia.
